# Is TAK1 a Direct Upstream Kinase of AMPK?

**DOI:** 10.3390/ijms19082412

**Published:** 2018-08-15

**Authors:** Dietbert Neumann

**Affiliations:** Department of Pathology, CARIM School for Cardiovascular Diseases, Faculty of Health, Medicine and Life Sciences, Maastricht University, 6200 MD Maastricht, The Netherlands; d.neumann@maastrichtuniversity.nl; Tel.: +31-43-387-7167

**Keywords:** TAK1, AMPK, phosphorylation, AMPK kinase

## Abstract

Alongside Liver kinase B1 (LKB1) and Ca^2+^/Calmodulin-dependent protein kinase kinase 2 (CaMKK2), Transforming growth factor-β (TGF-β)-activated kinase 1 (TAK1) has been suggested as a direct upstream kinase of AMP-activated protein kinase (AMPK). Several subsequent studies have reported on the TAK1-AMPK relationship, but the interpretation of the respective data has led to conflicting views. Therefore, to date the acceptance of TAK1 as a genuine AMPK kinase is lagging behind. This review provides with argumentation, whether or not TAK1 functions as a direct upstream kinase of AMPK. Several specific open questions that may have precluded the consensus are discussed based on available data. In brief, TAK1 can function as direct AMPK upstream kinase in specific contexts and in response to a subset of TAK1 activating stimuli. Further research is needed to define the intricate signals that are conditional for TAK1 to phosphorylate and activate AMPKα at T172.

## 1. About AMPK and TAK1

This review addresses questions that are relevant for experts in the field already familiar with AMPK and TAK1. To begin with, I will not discuss whether AMPK and TAK1 are a disparate couple or ideal affiliates, but rather provide entry points for further reading, in case readers are in need of information about AMPK or TAK1. I will not go into any detail with AMPK, because this review is part of the Special Issue on AMPK. Moreover, multiple authors (repeatedly) reviewed AMPK. In addition, there is a growing base of reviews focusing on different aspects of AMPK, such as the functions of AMPK in various tissues, or (patho-)physiological contexts (e.g., [1,2,3,4]). For AMPK novices, Hardie provides an excellent overview (e.g., [5,6,7,8]). In a nutshell, AMPK is an energy-sensing kinase that functions to maintain cellular and whole body energy balance [9]. AMPK is part of a protein kinase cascade [10]. T172 phosphorylation of the AMPKα subunit activates the kinase, which is dependent on upstream kinases, called AMPK kinases, identified as LKB1 and CaMKK2 [11,12,13,14].

TAK1 has been proposed as an alternative third AMPK kinase, which has received varying appreciation. This is the topic of this review. TAK1 is a serine/threonine protein kinase of the mitogen-activated protein kinase kinase kinase (MAP3K) family, playing a crucial role in regulating cell survival, differentiation, apoptosis, and inflammatory responses [15,16]. It forms complexes by binding to its accessory subunits, the TAK1-binding proteins (TAB1, TAB2, TAB3). TAK1 is activated by interleukin-1 (IL-1) and TGF-β receptors, tumour necrosis factor (TNF)-α, Toll-like receptors (TLR), CD40, and the B cell receptor. TAK1 is also involved in activating several intracellular kinases, p38 mitogen-activated protein kinase (p38MAPK), c-Jun N-terminal kinase (JNK), and IκB kinase complex (IKK). Therefore, TAK1 has been described as a regulator of nuclear factor κ-light-chain-enhancer of activated B cells (NF-κB) and MAPKs in proinflammatory signalling. More recently, this picture has been significantly amended, with the roles of TAK1 in tissue homeostasis (reviewed in [17]), as also further discussed below.

## 2. The Origin of the Debate

In 2006, after the discovery of LKB1 and CaMKK2 as upstream kinases of AMPK, TAK1 was identified as the third kinase capable of activating AMPK [18]. However, different from LKB1 and CaMKK2, TAK1 to date remains a disputed AMPK activating kinase. The reactions of the scientific community range from complete ignorance, to questioning TAK1 as an AMPK kinase, to acceptance without question. In this review, I will provide an overview on TAK1, with respect to its (putative) role as (direct) upstream activating kinase of AMPK.

In yeast, three alternative upstream kinases (Sak1, Tos3, and Elm1) have been described to activate the AMPK ortholog Snf1; knockout of all three kinases replicates the Snf1 knockout phenotype [19,20]. In search for alternative AMPK activating kinases in mammalian cells and by applying a screening approach in yeast, TAK1 was identified as in vitro upstream kinase of AMPK [18]. The in vivo relevance remained unknown, since the authors based their conclusion solely on cell-free and cell-based approaches. Notably, the study included evidence for TAK1 action on AMPK in LKB1-deficient HeLa cells. In the same year, cardiac-specific dominant-negative TAK1 mice were reported to show Wolff-Parkinson-White (WPW)-like phenotype [21], i.e., consistent with the idea that AMPK loss-of-function mutations in AMPKγ2 underlie WPW [22]. In the same study, TAK1 knockout embryos were shown to exhibit defective AMPK signalling. Due to observed midgestation embryonic lethality, the authors subsequently went on to acutely knock out floxed TAK1 alleles in cells using virally delivered Cre. The obtained results again generally supported a role of TAK1 upstream of AMPK, but the authors concluded that LKB1 could have been the intermediate of TAK1 action. The reason for this reservation was that acute loss of TAK1 interfered with the kinase activity of adenovirally delivered LKB1 complex, i.e., consisting of LKB1, mouse protein 25 (MO25) and STE20-related kinase adapter protein (STRAD). It should be noted that LKB1 is considered to be constitutively active upon complex formation with MO25 and STRAD [23,24]. Therefore, the mechanism of LKB1 inhibition, as observed by Xie et al., remains elusive. Accordingly, from these two early publications some discrepancy on the role of TAK1 upstream of AMPK primarily evolved around LKB1, and whether or not it mediates TAK1 effects on AMPK [18,21]. On the other hand, both reports agree on TAK1 as an important regulator of AMPK. In subsequent work on upstream kinases of AMPK, almost all studies have dealt with LKB1 and CaMKK2, which are firmly confirmed without any question. In contrast, the role of TAK1 as direct or indirect AMPK kinase remained obscure. 

Until today only few studies further addressed TAK1-AMPK signalling, of which the majority applied chemical tools (such as kinase inhibitors) that are prone to misinterpretation because of possible off-target effects. Moreover, a few reports also indicate signalling of AMPK to TAK1, i.e., turning AMPK into a possible activating kinase of TAK1 [25,26]. In this review, I am focusing on studies using genetic tools and offering clues on the exact role of TAK1 upstream of AMPK, but will also try to integrate controversial findings. As a guide to the reader, I am asking specific remaining open questions that are subsequently either partly or wholly answered, based on scientific evidence.

## 3. Is TAK1 Capable of Directly Phosphorylating AMPKα at T172 in Cell Free Assays?

In the original paper, Momcilovic et al. used a purified GST-fusion of the isolated Snf1 kinase domain that was directly incubated with an artificial construct of TAK1-TAB1 fusion protein purified from insect cells. Snf1 kinase domain was phosphorylated by a TAK1-TAB1 fusion protein at T210; the site equivalent to T172 in AMPK [18]. In my lab a bacterial co-expression strategy for TAK1 with TAB1 or with TAB2 was developed [27]. TAK1-TAB1 (but not TAK1-TAB2) was active upon co-expression in bacteria, strongly suggesting that the formation of TAK1-TAB1 complexes is sufficient for kinase activation. In contrast, recombinant AMPK heterotrimers after purification from bacteria are not phosphorylated in the α-subunit at T172 [28], but received this modification in presence of either LKB1-MO25-STRAD or TAK1-TAB1 complexes [23,27]. Therefore, mammalian AMPK heterotrimeric complexes can be directly activated by human TAK1-TAB1 complexes, in a process not requiring but resembling LKB1 complex, as shown in cell-free systems.

## 4. Is TAK1 Activating Cellular AMPK in Absence of LKB1?

This question has been already been addressed in the original work of Momcilovic et al., by using LKB1-deficient HeLa cells transfected with TAK1 and TAB1 plasmids, i.e., revealing that AMPK is activated by the wild-type but not the kinase defective TAK1-TAB1 complex [18]. In my lab, we obtained similar results in HeLa cells using wild type and mutant AMPK [27]. This approach rules out that TAB1 scaffolding is sufficient for AMPK activation. This is an important detail, because TAB1 has been shown to activate p38MAPK employing an unusual autophosphorylation mechanism [29]. In fact, even before TAK1 was suggested as a new upstream kinase, TAB1 was shown to co-immunoprecipitate with AMPK in cardiomyocytes [30]. This latter study also suggested that the association of AMPK with TAB1 did not require prior AMPK activation. Therefore, it seems unlikely that LKB1 action is needed for AMPK-TAB1 interaction, and TAB1 should be able to recruit TAK1 independent of LKB1. Further direct evidence for TAK1-AMPK signalling came from an unexpected pathway: in TNF-related apoptosis-inducing ligand (TRAIL)-treated epithelial cells AMPK was activated by TAK1 [31]. In this study, LKB1 and CaMKK2 were knocked down without affecting the ability of TRAIL to activate AMPK. In contrast, siRNA against TAK1, along with over-expression of kinase-defective TAK1 efficiently interfered with AMPK activation. These data establish TAK1-dependency, as well as LKB1-independency, at least in this particular setting.

## 5. Is Stimulation of TAK1 Sufficient for Activation of AMPK?

Many researchers doubt whether TAK1 can be considered a genuine upstream kinase, if TAK1 activation is seen in situations where AMPK is not activated. Indeed, Herrero-Martin et al. also observed that TNF-treatment activates TAK1 (as seen by IκB phosphorylation), but did not activate AMPK [31]. In addition, TAK1 activation originally was seen as an intracellular mediator of pro-inflammatory signals (such as TNF-α), giving rise to the development of TAK1 inhibitors for possible treatment of inflammatory disorders [32]. This prevalent view compounded scepticism about TAK1 as a genuine AMPK kinase, since reported AMPK effects are summarized to be the inverse, i.e., anti-inflammatory [3]. Therefore, the role of TAK1 as an upstream kinase of AMPK may be relevant only in certain physiological situations, or in response to specific signals. It should also be noted that LKB1 and CaMKK2 do not share the same input, and may well be active in situations where AMPK is not. In particular, LKB1, a constitutively active kinase upon complex formation, more efficiently phosphorylates AMPK in response to a drop in cellular energy level (through allosteric regulation of AMPK by AMP and ADP). CaMKK2 may be dependent on extracellular input (operating downstream of G-protein coupled receptors), but it is not clear whether transient Ca^2+^ waves, such as those occurring in contracting myocytes, are sufficient to activate AMPK in this cell type. However, both signals, LKB1 and CaMKK2 may also act synergistically [33]. Thus, AMPK phosphorylation at T172 increases through different pathways, downstream of various signals that can be intra- or extracellular. If TAK1 activation per se is insufficient for AMPK activation, TAK1 may still activate AMPK conditionally in response to specific upstream signals. 

## 6. What Is the Cellular Condition Where TAK1 Acts as an Upstream Kinase of AMPK?

In several recent studies, a possible role of TAK1 as upstream mediator of AMPK activation was verified by applying genetic knockdown strategies [34,35,36,37,38,39]. Although not verifying the role of TAK1 as a direct AMPK kinase, this approach puts TAK1 as an upstream AMPK activating signal into various cellular contexts. Moreover, TRAIL is an example of a distinct extracellular signal that activates TAK1-AMPK signalling [31]. Thus, the question may be asked, whether we can recognise a pattern of cellular challenges or signals where TAK1 is acting as activating AMPK kinase.

In recent literature, TAK1 is interpreted as a regulator of cell death and survival [17], which is well in accordance with the known functions of death ligands, such as TNFα and TRAIL [40]. Notably, TRAIL-induced TAK1-AMPK signalling was shown to induce cytoprotective autophagy in untransformed cells [31], whereas TRAIL induces apoptosis in several cancer cell types. Autophagy is a survival mechanism, which can be elicited by various sublethal stresses as a response to fluctuating external conditions, ranging from extracellular signals, to a change in pH, temperature or oxygen tension [41]. Some of these stresses are not predicted to directly affect cellular energy levels. The known role of AMPK in the control of autophagy in response to nutrient starvation is commonly linked to LKB1 signalling, whereas TRAIL elicits autophagy via TAK1-AMPK [31]. Of note, independent of the upstream signalling pathway, autophagy is an important survival mechanism, providing the cell with building blocks and metabolites. This integrates well with one of AMPKs more general roles; limiting cell proliferation and growth, as well as energy expense, in times of nutrient scarcity, while also enhancing the cell’s ability to survive stresses, such as hypoxia and glucose deprivation [42].

As already indicated, TAK1 was shown to activate AMPK in response to various stimuli and different cell types. Receptor activator of NF-κB ligand (RANKL) activated AMPK in osteoclast precursors, and siRNA-mediated TAK1 knockdown blocked RANKL-induced activation of AMPK [34]. RANKL is a member of the TNF superfamily, supporting the idea that a subset of TAK1 activating signals could physiologically activate AMPK. In endothelial cells, Vascular endothelial growth factor (VEGF) stimulated TAK1 and AMPK, whereas TAK1 downregulation by shRNA also inhibited VEGF–stimulated phosphorylation of several kinases, including AMPK [36].

Belinostat promoted reactive oxygen species (ROS) production in PANC-1 cells and increased the ROS induced TAK1/AMPK association resulting in AMPK activation. Anti-oxidants, as well as TAK1 shRNA knockdown, suppressed Belinostat-induced AMPK activation and PANC-1 cell apoptosis [35].

Fasted mice deficient of TAK1 in hepatocytes exhibited severe hepatosteatosis with increased mTORC1 activity, and suppression of autophagy compared with their WT counterparts [43], suggesting reduced AMPK function in these livers. TAK1-deficient hepatocytes exhibited autophagy and suppressed AMPK activity in response to starvation or metformin treatment; however, ectopic activation of AMPK restored autophagy in these cells. These data indicate that TAK1 regulates hepatic lipid metabolism and tumorigenesis via the AMPK/mTORC1 axis [43]. Therefore, it was proposed that TAK1-mediated autophagy in the liver plays a role in preventing excessive lipid accumulation induced by starvation and fat overload [44]. Knockdown of TAK1 decreased the AMPK phosphorylation induced by overexpression of a dominant-negative form of p38α [38], which the authors interpreted as a negative feedback loop. Recent data suggested that TAK1 could be the upstream kinase for AMPK activation by *Helicobacter pylori*, since partial depletion of TAK1 by shRNAs not only inhibited AMPK activation, but also suppressed survival of *H. pylori*-infected gastric epithelial cells [37]. Activation of TAK1 was also found to restrict *Salmonella typhimurium* growth by inducing AMPK activation and autophagy [45]. In this study, TAK1 siRNA led to the inhibition of *S. typhimurium*-induced phosphorylation of AMPK T172, ULK1 S317, and ACC S79. The authors concluded that TAK1 activation leads to AMPK activation, which activates ULK1 by phosphorylating ULK1 S317 and suppressing mTOR activity and ULK1 S757 phosphorylation.

In conclusion, published data indicate TAK1-dependent AMPK activation could be required for induction of autophagy, as a possible survival mechanism in response to acute and specific life-threatening challenges. TAK1-induced autophagy may thus occur in the absence of an energy challenge, such as those elicited through extracellular factors (e.g., TRAIL), or bacterial infections (e.g., *H. pylori*, *S. typhimurium*), and oxidative stress (e.g., Belinostat).

Further conditions promoting TAK1-dependent AMPK activation are likely to be identified.

## 7. Does AMPK Have a Role in Activating TAK1?

AMPK has been reported to activate TAK1 and mediate pro-inflammatory effects in THP-1 cells [25]. In this study, it was shown that pro-inflammatory signals activated TAK1 signalling, which was then inhibited by AMPKα knockdown. Taking into account the ability of AMPK to bind TAB1 [21], and considering the role of TAB1 in activating TAK1, the interpretation of AMPK as upstream kinase of TAK1 could consequently be challenged. For, example, could the lack of AMPK reduce the availability of TAB1 for subsequent activation of TAK1? Notably, binding of TAB1 to TAK1 in a sequence of molecular events, activates TAK1 by autophosphorylation of T184/T187 [27], and does not require any upstream kinase. Interestingly, the authors of the same study confirmed AMPK-TAK1 interaction in their model, which required both the AMPKα autoinhibitory-domain, and the TAB1-binding domain of TAK1 [25]. The possible AMPK-TAB1 complex formation, and putative requirement of TAB1 as a mediator of AMPK-TAK1 binding in THP-1 cells was not investigated.

In another recent study, AMPKα1 was suggested to participate in renal TAK1 activation and TAK1-dependent signalling induced by angiotensin-II [26]. Angiotensin-II increased the phosphorylation of TAK1 (S412) in renal tissue of AMPKα1+/+ mice but not AMPKα1−/− mice. Notably, S412 is targeted by PKA [46]. Furthermore, the authors also observe that angiotensin-II upregulates the AMPKα1 isoform in renal tissue, and increased TAK1-target gene mRNA and renal protein expression in AMPKα1+/+ mice, but less-so in AMPKα1−/− mice [47]. Using the same argumentation as above, if AMPKα indeed acts as a scaffold for TAB1, one could predict that TAK1 activity is downregulated in AMPKα knockouts.

Therefore, AMPK may be involved in TAK1 activation, but not necessarily as an upstream kinase. Importantly, to date, there is only circumstantial evidence for AMPK to activate TAK1, whereas biochemical proof is available and functional data is accumulating to support TAK1 as a genuine direct AMPK activating kinase.

## 8. Conclusions

**Figure 1:**
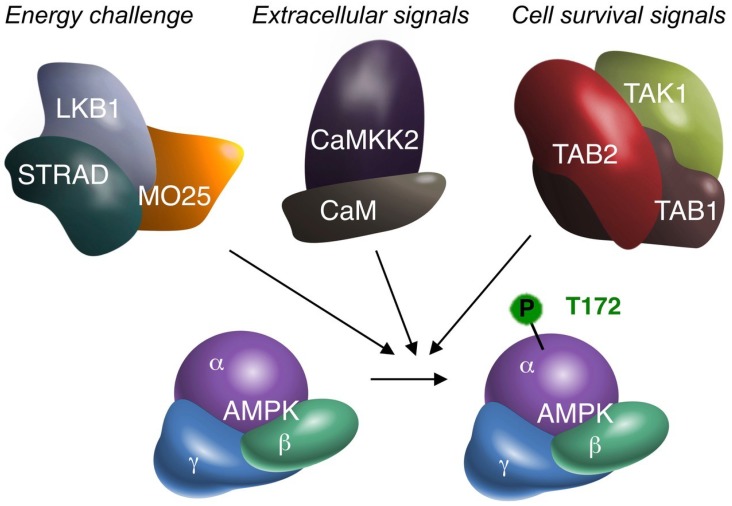
The three alternative AMPK kinases. Biochemical (cell-free), cell biological (in vitro) and animal (in vivo) experimentation suggest that TAK1 can activate AMPKα by phosphorylation of the critical T172 residue. Summative evidence therefore supports TAK1 as an additional AMPK upstream kinase, besides LKB1 and CaMKK2. AMPK may receive (simultaneous) activation from all three upstream kinases. The original signal leading to AMPK activation may differ per upstream kinase, as suggested above. All four kinases are depicted with their accessory subunits, as functional protein complexes. The requirement of TAB1/TAB2/TAB3 for AMPK activation has not been fully elucidated. However, to date TAB1 and/or TAB2 are the most likely candidates. TAB1 may also bind to AMPK independent of TAK1 [30]. MO25: mouse protein 25; STRAD: STE20-related kinase adapter protein; CaM: Calmodulin.

About 12 years after the original publication reporting TAK1 as a ‘candidate’ AMPK kinase [1], as argued above, the collective data rather confirms the suggested authentic role. Thus, I propose to accept TAK1, in addition to LKB1 and CaMKK2, as the third genuine upstream kinase of AMPK (Figure 1).

## Abbreviations

AMPK	AMP-activated protein kinase
CaMKK2	Ca^2+^/Calmodulin-dependent protein kinase kinase 2
LKB1	Liver kinase B1
T172	Threonine 172 residue (of AMPKα)
TAB1	TAK1 binding protein 1
TAB2	TAK1 binding protein 2
TAB3	TAK1 binding protein 3
TAK1	Transforming growth factor β-activated protein kinase
TNF-α	Tumour necrosis factor α
TRAIL	Tumor necrosis factor related apoptosis inducing ligand
